# Comprehensive Analysis of Whole-Transcriptome Profiles in Response to Acute Hypersaline Challenge in Chinese Razor Clam *Sinonovacula constricta*

**DOI:** 10.3390/biology12010106

**Published:** 2023-01-10

**Authors:** Wei Cao, Yinghui Dong, Yusong Geng, Siqi Bi, Zhihong Liu, Liqing Zhou, Xiujun Sun, Sudong Xia, Changfeng Chi, Biao Wu

**Affiliations:** 1 Key Laboratory of Sustainable Development of Marine Fisheries, Ministry of Agriculture and Rural Affairs, Yellow Sea Fisheries Research Institute, Chinese Academy of Fishery Sciences, Qingdao 266071, China; 2 National and Provincial Joint Engineering Research Centre for Marine Germplasm Resources Exploration and Utilization, School of Marine Science and Technology, Zhejiang Ocean University, Zhoushan 316022, China; 3 Key Laboratory of Aquatic Germplasm Resources of Zhejiang, Zhejiang Wanli University, Ningbo 315100, China; 4 Key Lab of Aqua-Ecology and Aquaculture, Department of Fishery Science, Tianjin Agricultural University, Tianjin 300384, China; 5 Laboratory for Marine Fisheries Science and Food Production Processes, Qingdao National Laboratory for Marine Science and Technology, Qingdao 266273, China

**Keywords:** high salt stress, non-coding RNA, ceRNA, transcriptome, *Sinonovacula constricta*

## Abstract

**Simple Summary:**

Salinity can affect the physiological, reproductive, and developmental characteristics of marine molluscs. The Chinese razor clam (*Sinonovacula constricta*) is a marine bivalve that inhabits estuarine and intertidal habitats where salinity is usually lower when compared to open sea waters. The species is also an economically important subject of aquaculture in China. Here, RNA sequencing was used to study the internal mechanism of salt stress response in the Chinese razor clam. Differentially expressed RNAs were identified and lncRNA/circRNA-miRNA-mRNA regulatory networks were successfully constructed. Furthermore, the expression of four candidate genes in the Chinese razor clam subjected to different salinity stress were examined by qRT-PCR. The results of this study will improve our understanding of genetic-level responses to salinity changes in the Chinese razor clam. Moreover, the acquired information may be useful for optimization of the artificial breeding of the species under aquaculture conditions.

**Abstract:**

The Chinese razor clam (*Sinonovacula constricta*) is an important for Chinese aquaculture marine bivalve that naturally occurs across intertidal and estuarine areas subjected to significant changes in salinity level. However, the information on the molecular mechanisms related to high salinity stress in the species remain limited. In this study, nine gill samples of *S. constricta* treated with 20, 30, and 40 ppt salinity for 24 h were used for whole-transcriptome RNA sequencing, and a regulatory network of competing endogenous RNAs (ceRNAs) was constructed to better understand the mechanisms responsible for adaptation of the species to high salinity. A total of 83,262 lncRNAs, 52,422 mRNAs, 2890 circRNAs, and 498 miRNAs were identified, and 4175 of them displayed differential expression pattern among the three groups examined. The KEGG analyses of differentially expressed RNAs evidenced that amino acid synthesis and membrane transport were the dominant factors involved in the adaptation of the Chinese razor clam to acute salinity increase, while lipid metabolism and signaling played only a supporting role. In addition, lncRNA/circRNA-miRNA-mRNA regulatory networks (ceRNA network) showed clearly regulatory relationships among different RNAs. Moreover, the expression of four candidate genes, including tyrosine aminotransferase (*TAT*), hyaluronidase 4 (*HYAL4*), cysteine sulfinic acid decarboxylase (*CSAD*), and ∆^1^-pyrroline-5-carboxylate synthase (*P5CS*) at different challenge time were detected by qRT-PCR. The expression trend of *TAT* and *HYAL4* was consistent with that of the ceRNA network, supporting the reliability of established network. The expression of *TAT*, *CSAD*, and *P5CS* were upregulated in response to increased salinity. This might be associated with increased amino acid synthesis rate, which seems to play an essential role in adaptation of the species to high salinity stress. In contrast, the expression level of *HYAL4* gene decreased in response to elevated salinity level, which is associated with reduction Hyaluronan hydrolysis to help maintain water in the cell. Our findings provide a very rich reference for understanding the important role of ncRNAs in the salinity adaptation of shellfish. Moreover, the acquired information may be useful for optimization of the artificial breeding of the Chinese razor clam under aquaculture conditions.

## 1. Introduction

Salinity is an important ecological factor that influences the distribution, physiology, reproduction, and development of aquatic animals [1,2]. Compared to relatively stable salinity level of open sea waters, intertidal zones, estuaries, and land-based seawater ponds are characterized by a high variation in water salinity [3,4,5]. Therefore, marine organisms inhabiting the intertidal zone and estuaries must be physiologically able for adaptation to salinity changes. It is necessary to understand molecular mechanisms involved in the salinity change response in marine organisms to predict future consequences of advancing alterations in water salinity of intertidal and estuarine habitats caused by climate change. Moreover, it can provide useful information for the aquaculture production of marine organisms.

At present, high-throughput sequencing technology has found an application in the exploration the genetic mechanisms underlying salinity response. Beside mRNAs, many non-coding RNAs (ncRNAs), previously regarded as junk molecules, have also been found to play a role in the regulation of many physiological responses [6]. NcRNA is a type of RNA transcribed through regions of genome that does not code for amino acids. It includes microRNA (miRNA), long non-coding RNA (lncRNA), and circular RNA (circRNA) [6]. In human, at least 75% of the genome is transcribed into ncRNAs [7], which play pivotal roles in multiple cellular physiological and pathological processes [8]. Increasing evidence has also shown that these ncRNAs could participate in osmotic regulation [9,10,11,12,13,14,15]. So far, most studies on aquatic organisms have been reported on miRNAs. For example, the miRNAs in swimming crab (*Portunus trituberculatus*) have been explored and enriched into six important GO biological processes related to osmoregulation, such as anion transport and the chitin metabolism [16]. The miR-10 agomir or antagomir of sea cucumber (*Apostichopus japonicas*) has been found to downregulate the expression of TBC1D5 [17]. However, the studies on lncRNAs and circRNAs are very limited. It has been shown that lncRNAs and circRNAs can be used as “sponges” for miRNAs to counteract the inhibition of miRNA-mediated mRNAs by competing for endogenous RNA (ceRNA) networks [18]. Therefore, the study on lncRNAs and circRNAs can provide more essential information for revealing the mechanisms underlying the physiological processes.

The Chinese razor clam (*Sinonovacula constricta*) is a marine bivalve that naturally occurs in the estuary and intertidal regions where salinity is usually lower than in open sea. As one of the four traditional shellfish for aquaculture in China, the Chinese razor clam is an economically important species, produced in large scale along the coastal areas from Liaoning to Fujian province [19]. Since greater ecological benefits and higher economic income, integrated pond culture has recently become a popular mode for the Chinese razor clam aquaculture production in northern China. However, water salinity conditions in pond may change greatly due to external environmental factors including drought, high temperature, etc. Although the species can withstand salinity stress to some extent, the acute increase in salinity often leads to large-scale mortality [20]. Recent studies on salinity adaptation have mainly focused on the effects of salinity on individual growth rate, development, and metabolism [19,21]. However, only few studies have explored the molecular mechanisms underlying the Chinese razor clam’s response to salinity stress. To our best knowledge, none of them included information on the ncRNA regulation mechanism in response to salinity changes in the species.

Therefore, in this study, the effects of high salt stress on the expression of mRNAs and ncRNAs of in the Chinese razor clams were explored. The results of this study will improve our understanding of genetic level responses to salinity changes in the Chinese razor clam. Moreover, the acquired information may be useful for optimization of the artificial breeding of the species under aquaculture conditions.

## 2. Materials and Methods

### 2.1. Experimental Samples

Healthy *S. constricta* with a shell length of 52.68 ± 2.77 mm were obtained from the Qingdao sea area (Shandong Province of China, 36.5900° N, 120.8024° E), where the annual salinity ranges from 18 ppt to 22 ppt. Before salinity challenge, the collected clams were acclimated for one week in an incubator containing aerated seawater with salinity of 20 ± 1 ppt, temperature of 25 ± 1 °C, pH of 7.75 ± 0.25, dissolved oxygen of 7.56 ± 0.23 mg/L, and ammonia < 0.02 mg/L. During acclimation, the Chinese razor clams were fed twice a day with *Isochrysis galbana*.

### 2.2. Salinity Challenge and Tissue Sampling

In this study, the Chinese razor clam individuals were divided into three groups, namely A (set as control), B, and C, which were treated with salinities of 20 ppt, 30 ppt, and 40 ppt, respectively. All other conditions were the same as during the acclimation period. Different salinities were obtained by diluting seawater with freshwater or adding sea salt (Guangzhou Yier Bioengineering Company, Guangzhou, China). The gill samples from three random individuals per each group were collected randomly at 0 h, 24 h, 48 h, and 96 h after exposure to changed salinity. The samples collected at 24 h were used for whole transcriptome sequencing and the samples collected at all time points were used for qRT-PCR. After collection, the samples were immediately frozen in liquid nitrogen and stored at −80 °C until further analyses.

### 2.3. RNA Sequencing

Total RNAs were isolated from the nine samples using TRIzol reagent (Invitrogen, Carlsbad, CA, USA) according to the manufacturer’s instructions. Nanodrop (Thermo Fisher, Shanghai, China) and Agilent LabChip GX 2100 (Beijing, China) were used to evaluate the purity, concentration, and integrity of extracted RNA.

For mRNA, lncRNA, and circRNA sequencing, a Ribo-Zero rRNA Removal Kit (Epicentre, Madison, WI, USA) was used to remove ribosomal RNAs (rRNA) from the extracted RNA. After removing rRNA, about 1.5 μg of input RNA were fragmented by divalent cations under a high temperature. The random hexamer primers and reverse transcriptase were used for first-strand cDNA synthesis. DNA Polymerase I and RNase H were used for second-strand cDNA synthesis. For small RNA sequencing, nine small RNA libraries were constructed with 3 μg of total RNA, using the VAHTSTM Small RNA Library Prep Kit (Illumina, San Diego, CA, USA). After library preparation, template concentration and insert size were determined using Qubit 3.0 and Agilent LabChip GX 2100, respectively. Finally, all the verified libraries were sequenced using an Illumina NovaSeq 6000 platform (Illumina, San Diego, CA, USA).

### 2.4. Identification of RNAs

After sequencing quality control, the clean reads were aligned to the Chinese razor clam genome with HISAT2 [22] to obtain mapped reads, which were assembled to transcripts using StringTie [23]. After discarding transcripts with FPKM (fragments per kilobase of transcript per million mapped reads) < 0.5, mRNAs and RNAs owning potential coding capacity, the non-coding transcripts were identified as final lncRNAs. Four softwares including CPC2 [24], CNCI [25], CPAT [26], and Pfam [27] were used to screen for potential coding capacity of RNAs. The circRNAs were screened and identified within the constructed lncRNA library by using CIRI [28]. First, CIRI took 20 bp as the anchor point at both ends of the reads on the genomic alignment, then used the BMA-MEM [29] algorithm to compare with the reference genome to generate a SAM file, after which the paired chiastic clipping signals were scanned from the SAM file. If the sequences on both sides possess GT/AG splicing signals, respectively, they would be considered as circRNAs. The miRNAs were identified by aligning the sequences of the mapped reads with the sequences of the mature miRNAs in miRBase (v22). If the read sequences were fully identical to those deposited in the database, they were considered as known miRNAs. The novel miRNAs were predicted by using software miRDeep2 software [30]. Possible precursor sequences were obtained by comparing reads to position information on the genome. According to the distribution information of reads on the precursor sequences, the Bayesian model was used to finally predict new miRNAs.

### 2.5. Target Gene Prediction

The adjacent genes within 100 kb upstream and downstream of lncRNAs were recognized as cis-target genes of lncRNAs by Perl script. The correlation coefficient between lncRNA and mRNA greater than 0.9 was used as criterion to screen and predict lncRNA trans-acting target genes. The software MiRanda [31] and Targetscan [32] were used to predict the target genes of miRNAs, as well as to find miRNA binding sites in the 3′UTR region of genes and 1000 bp downstream of the stop codon for genes without the 3′UTR region. Since circRNA contains multiple miRNA binding sites, the method of miRNA target gene prediction was used to identify the miRNA binding sites in circRNA, and the function of circRNA was clarified according to the functional annotation of miRNA target gene.

### 2.6. GO and KEGG Analysis of Differentially Expressed RNAs

The edgeR software package was used to detect differentially expressed genes (DEGs), differentially expressed lncRNA (DELs), differentially expressed circRNA (DECs) and differentially expressed miRNA (DEMis) with *p* < 0.01 and |log_2_FoldChange| ≥ 1.5 [33]. The ToGO R [34] and KOBOS software [35] were used to perform GO enrichment analysis and KEGG enrichment analysis of DEGs and ncRNA target genes, respectively.

### 2.7. Construction of the lncRNA/circRNA-miRNA-mRNA Network

The miRanda software was used to analyze interactions between DEMis and DEGs, and between DELs and DECs [36]. Cytoscape V3.9 software was used to visualize the potential lncRNA/circRNA-miRNA-mRNA regulatory network, and expression trends annotated as “up-down-up” or “down-up-down” ceRNA networks were selected for further research [37].

### 2.8. Quantitative Real-Time PCR Validation

To verify the sequencing results, 16 differentially expressed RNAs including four mRNAs (*UBR4*, *TAT*, *Gal9*, and *SR*), seven lncRNAs, two circRNAs, and three miRNAs were selected for qRT-PCR validation, with the *RS9* [38] (for mRNA, lncRNA, and circRNA) and U6 [39] (for miRNA) being set as the internal reference genes. For the qRT-PCR of mRNA, lncRNA, and circRNA, cDNA templates were synthesized by using HiScript III RT SuperMix for qPCR (+gDNA wiper) (Vazyme, China). For the qRT-PCR of miRNA, cDNA templates were synthesized by MonScriptTM miRNA First Strand Cdna Synthesis Kit (Tailing Reaction) (Monad, China).

Among identified DEGs, four genes that displayed the most pronounced response to salinity change, i.e., (1) cysteine sulfinic acid decarboxylase (*CSAD*, the rate-limiting enzyme of the taurine synthesis pathway), (2) ∆^1^-pyrroline-5-carboxylate synthase (*P5CS*, the rate-limiting enzyme of the proline synthesis pathway), (3) tyrosine aminotransferase (*TAT*, up-regulated in the ceRNA networks) and (4) hyaluronidase 4 (*HYAL4*, down-regulated in the ceRNA networks), and each were selected for qRT-PCR analysis.

The qRT-PCR amplification was performed using the CFX connect real-time PCR system (Bio-Rad, USA). Relative gene expression levels were calculated using the 2^−∆∆ct^ method. According to the gene sequence, Primer Premier 5.0 was used to design specific primers for the qRT-PCR, and all primers used in the analysis are listed in Table 1.

## 3. Results

### 3.1. Sequencing Data

For mRNA, lncRNA, and circRNA sequencing, nine libraries produced a total of 157.66 GB clean data, with more than 15.33 GB per sample and the percentages of Q30 bases above 89.87%. The clean reads of each sample were mapped separately to the designated reference genome, and the matching rate ranged from 59.38% to 69.53%. After small RNA sequencing, nine samples yielded 113.66 MB clean reads, with more than 10.57 MB per sample and the percentage of Q30 bases higher than 92.91%. The matching efficiency ranged from 52.43% to 67.30%. In all, 83,262 lncRNAs, 52,422 mRNAs, 2890 circRNAs, and 498 miRNAs were identified in the nine samples (Figure S1).

### 3.2. Expression Pattern of mRNA under High Salinity Stress

**Figure 1:**
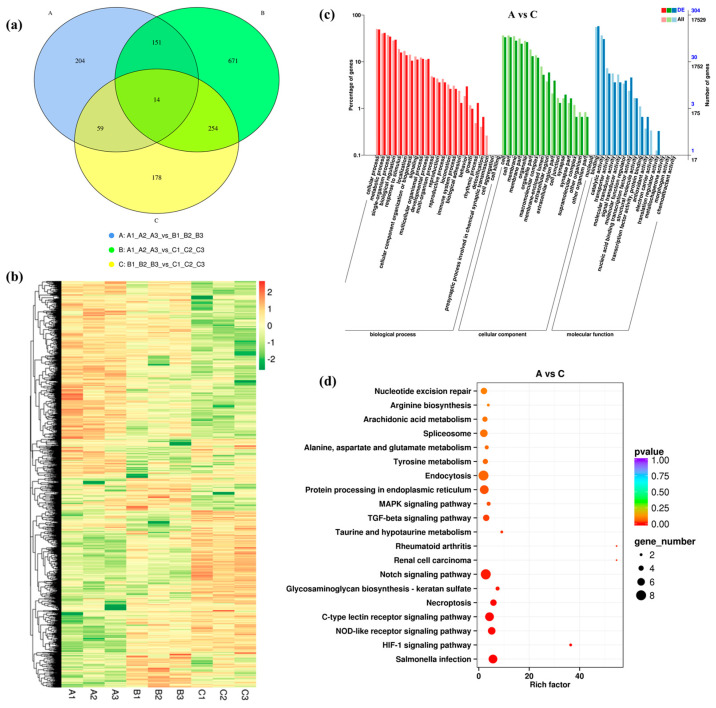
Identification and analysis of DEGs under salinity stress. (**a**) Venn diagram showing the number of DEGs in groups A vs. B, A vs. C and B vs. C. (**b**) Heat map of all DEGs. (**c**) GO enrichment analysis of DEGs in groups A vs. C. (**d**) KEGG enrichment analysis of DEGs in groups A vs. C.

From RNA-seq data, we identified 428 DEGs in groups A vs. B (245 up-regulated and 183 down-regulated), 1090 DEGs in groups A vs. C (494 up-regulated and 596 down-regulated) and 505 DEGs in groups B vs. C (286 up-regulated and 219 down-regulated). Their log2FC values ranged from −12.10 to 12.94 (Figure S2). Moreover, 14 DEGs were common among A vs. B, A vs. C, and B vs. C, implying that they might participate in the basic response process under salinity challenge (Figure 1a). The hierarchical clustering of 1531 DEGs in the heatmap showed that the three repeats of each treatment were always clustered together, suggesting good reproducibility (Figure 1b).

GO annotation for DEGs showed that the “cellular process” and “metabolic process” were the dominant annotated biological process (BP) (Figure 1c and Figure S3). In groups A vs. C more DEGs were annotated to “response to single-organism process” under BP when compared to groups A vs. B and B vs. C. For cellular component (CC), between A vs. C groups most DEGs (102) were annotated to “cell”, while between A vs. B groups majority of DEGs (N = 64) were annotated to “membrane”. In addition, a large amount of DEGs across all groups were annotated to “organelle”, “cell part” and “membrane part”. Notably, for molecular function (MF), most DEGs were annotated to “binding”, “catalytic activity” and “transporter activity”.

KEGG enrichment analysis was performed to determine further metabolic pathways. The top 20 pathways with the lowest significant q values are shown as Figure 1d and Figure S3. Among them, the top enriched pathways for DEGs in groups A vs. B, A vs. C, and B vs. C were “Endocytosis”, “Notch signaling pathway”, and “C-type lectin receptor signaling pathway”, respectively. In group A vs. B, DEGs were mainly enriched in the pathways including “Endocytosis” (11 DEGs, 10 up-regulated and one down-regulated) and “Tyrosine metabolism” (six DEGs, three up-regulated and three down- regulated). In group A vs. C, they were mainly enriched in “Notch signaling pathway” (eight DEGs, two up-regulated and six down-regulated) and “Protein processing in endoplasmic reticulum” (seven DEGs, six up-regulated and one down-regulated). In turn, within group B vs. C, they were enriched in “C-type lectin receptor signaling pathway” (six DEGs, four up-regulated and two down-regulated) (Table S1).

Within screened DEGs some important potential genes related to osmoregulation were also identified (Table S2). Among them ABC transporter G family member 14 (*ABCG14*), solute carrier family 25 (*SLC25*), and *CSAD* that are related to ion transporter and amino acid biosynthesis genes, were up-regulation in response to increased salinity. Moreover, some stress genes such as heat shock proteins and E3 ubiquitin protein ligase and related to immune system, such as galectin, C-type lectin, and C1q domain-containing proteins were also found to be involved in the response to salinity stress. Interestingly, we also found changes in the expression of many other genes encoding biological enzymes.

### 3.3. Expression Pattern of lncRNA under High Salinity Stress

**Figure 2:**
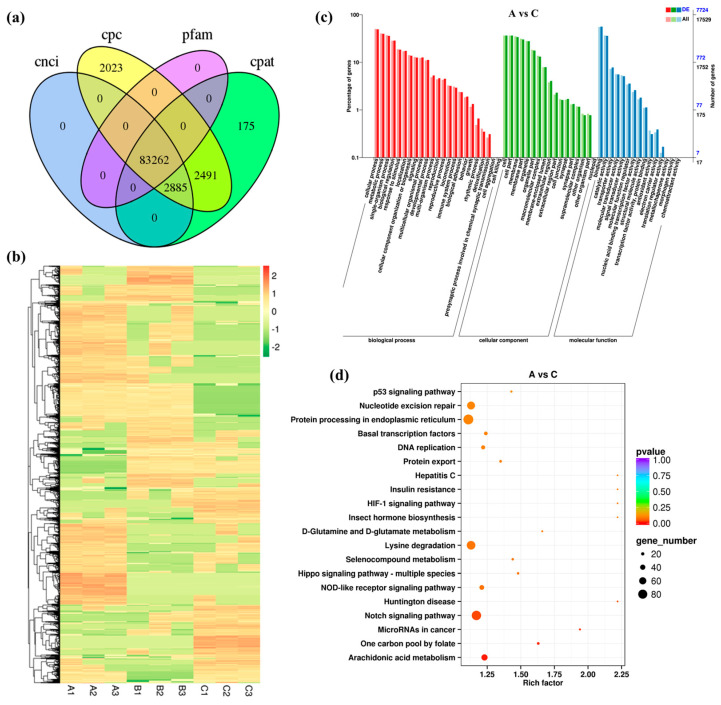
Identification and analysis of DELs under salinity stress. (**a**) Venn diagram showing the number of lncRNAs identified by CNCI, CPC, Pfam, and CPAT methods. (**b**) Heat map of all DELs. (**c**) GO enrichment analysis of DELs in groups A vs. C. (**d**) KEGG enrichment analysis of DELs in groups A vs. C.

A total of 83,262 lncRNAs were identified by CNCI, CPC, CPAT, and Pfam analysis (Figure 2a). Through comparative analysis, 818 DELs in group A vs. B (308 up-regulated and 510 down-regulated), 1240 DELs in group A vs. C (481 up-regulated and 759 down-regulated) and 759 DELs in group B vs. C (320 up-regulated and 439 down-regulated) were identified (Figure S4). Hierarchical clustering of 2317 DELs in the heatmap showed that the three repeats of each treatment were always clustered together (Figure 2b). A large number of DELs were highly expressed in the group subject to high salinity stress. These recorded results evidenced that the lncRNAs were also involved in the responses of the Chinese razor clam to salinity stress.

To investigate the possible function of lncRNAs in osmoregulation, their cis- and trans-targeted mRNAs were predicted with bioinformatics methods. The GO annotation of DELs and DEGs led to almost the same results (Figure 2c and Figure S5). KEGG analysis revealed that the top one enriched pathways for the target genes of DELs in groups A vs. B, A vs. C, and B vs. C were “Arginine and proline metabolism”, “Arachidonic acid metabolism”, and “ABC transporters”, respectively (Figure 2d and Figure S5). The two most significantly enriched pathways were “Neuroactive ligand-receptor interaction” and “Ubiquitin mediated proteolysis” (Table S3), with both involving more than 100 genes. Furthermore, the target genes of 68 DELs in groups A vs. C were enriched in Carbon metabolism (Table S3). These findings contribute to our understanding of how lncRNAs are involved in the regulation of hyperosmolarity.

### 3.4. Expression Patterns of circRNAs under High Salinity Stress

**Figure 3:**
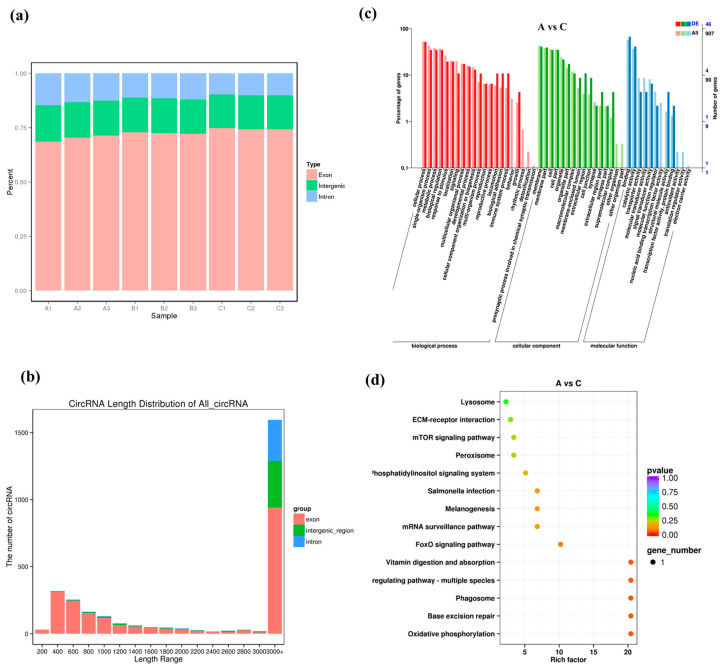
Identification and analysis of DECs under salinity stress. (**a**,**b**) Type distribution and Sequence length of all identified circRNAs. (**c**) GO enrichment analysis of DECs in groups A vs. C. (**d**) KEGG enrichment analysis of DECs in groups A vs. C.

In total, 2890 circRNAs were identified, and about 70%, 20%, and 10% of them belonged to exon, intergenic region, and intronic type, respectively (Figure 3a). The sequence length distribution of circRNAs is shown in Figure 3b, and most of them were longer than 3000 bp or shorter in a range of 400–600 bp. We identified 101 DECs in group A vs. B (45 up-regulated and 56 down-regulated), 98 in groups A vs. C (44 up-regulated and 54 down-regulated) and 89 in group B vs. C (46 up-regulated and 43 down-regulated), and their log2FC values are presented as Volcano Plot in Figure S6.

The GO enrichment for DECs indicated the “cellular process” and “single-organism process” were the dominant in BP term. For CC and MF, most DECs were annotated to “membrane” and “cell”, as well as “binding” and “catalytic activity”, respectively (Figure 3c and Figure S7). The DECs in groups A vs. B, A vs. C and B vs. C were significantly enriched in 14, 14, and eight pathways in KEGG analysis, respectively (Figure 3d and Figure S7). Notably, some interesting pathways such as “glutathione metabolism”, “carbon metabolism”, “oxidative phosphorylation” and “lysosomal” were found.

### 3.5. Expression Pattern of miRNA under High Salinity Stress

**Figure 4:**
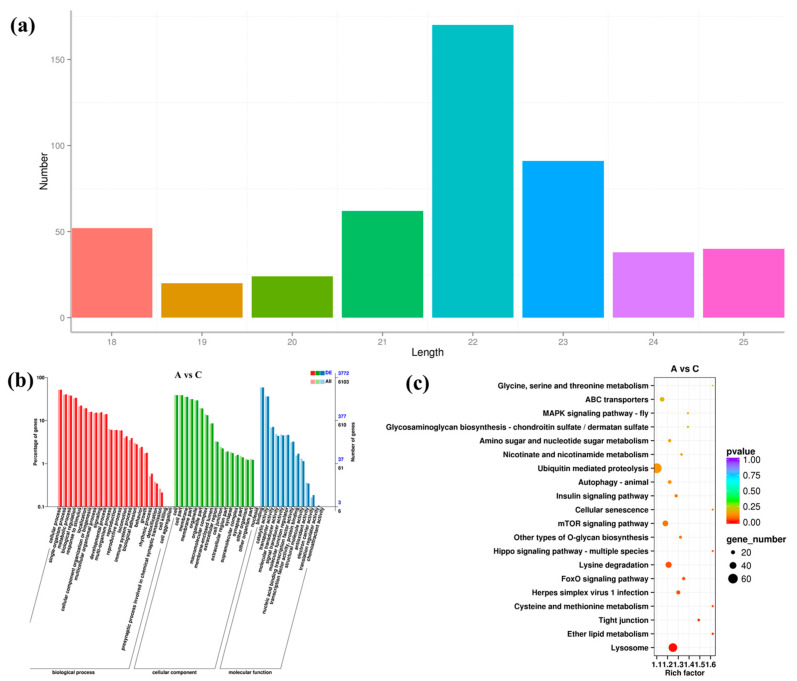
Identification and analysis of DEMis under salinity stress. (**a**) Sequence length of all identified miRNAs. (**b**) GO enrichment analysis of DEMis in groups A vs. C. (**c**) KEGG enrichment analysis of DEMis in groups A vs. C.

Small RNA-seq yielded a total of 498 miRNAs, and among them, 170 (34%) were 22 nt long and 92 (18%) were 23 nt long, together accounting for the majority of the identified miRNAs (Figure 4a). In addition, a total of 12,085 target genes were predicted for the 498 miRNAs. For most miRNAs, they were predicted to regulate multiple target genes. As shown in Table S4, a novel miRNA, namely, novel-miR-37, had 285 target genes. It showed there were 12 DEMis in group A vs. B (seven up-regulated and five down-regulated), 132 in group A vs. C (121 up-regulated and 11 down-regulated), and 98 in group B vs. C (86 up-regulated and 12 down-regulated), respectively (Figure S8). Clustering analysis still confirmed the good experimental repeatability (Figure S9).

Among the 12,085 target genes of DEMis, 6103 and 5619 were successfully enriched by GO and KEGG, respectively. GO results of DEMis were consistent with those of DEGs (Figure 4b and Figure S10). For the DEMis target genes of groups A vs. B, A vs. C, and B vs. C, the top one enriched pathways were “Hippo signaling pathway-mltiple species”, “Lysosome”, and “ABC transporters”, respectively (Figure 4c and Figure S10). In addition, “Glycerophospholipid metabolism” was enriched for the target genes of DEMis in groups B vs. C.

### 3.6. Construction of Potential lncRNA/circRNA-miRNA-mRNA Regulatory Networks

**Figure 5:**
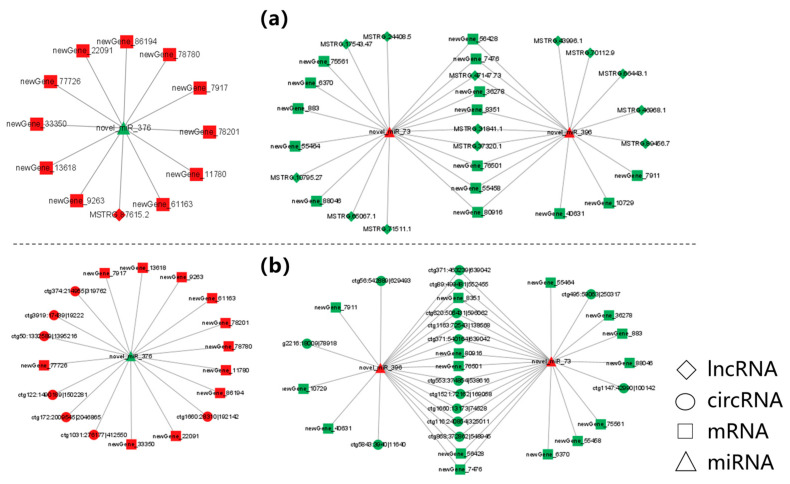
Interaction diagram of ceRNA network related to osmoregulation. (**a**) Parts of lncRNA-miRNA-mRNA regulatory network. (**b**) Parts of circRNA-miRNA-mRNA regulatory network. Red and green nodes represent up-regulated and down-regulated transcripts, respectively.

In this study, the lncRNA/circRNA-miRNA-mRNA networks were constructed based on the ceRNA theory. Those networks included two expression trends, namely “down-up-down” and “up-down-up”. A total of 99 mRNAs, 63 miRNAs, and 68 lncRNAs were screened and used to construct a lncRNA-miRNA-mRNA network, which included 4275 ligation sites. Moreover, a circRNA-miRNA-mRNA network was also constructed using 67 mRNAs, 69 miRNAs, and 21 circRNAs, and this network included 1147 ligation sites. As a representative demonstration, from the lncRNA/circRNA-miRNA-mRNAs network, the significantly up-regulated novel_miR_376 and significantly down-regulated novel_miR_396 and novel_miR_73 as well as their associated targeting mRNAs, lncRNAs, and circRNAs were selected for mapping analysis. The target genes were closely related to osmotic pressure regulation. It was found that those genes were enriched in many terms including “Tyrosine metabolism”, “deoxyribonucleotide biosynthetic process”, “integral component of membrane”, and “Glycosaminoglycan degradation”. These target genes especially included up-regulated *TAT*, as well as down-regulated G protein pathway suppressor 2 (*GPS2*) and *HYAL4*. Parts of two network schematic diagrams are shown in Figure 5.

### 3.7. Quantitative Real-Time PCR Validation

**Figure 6:**
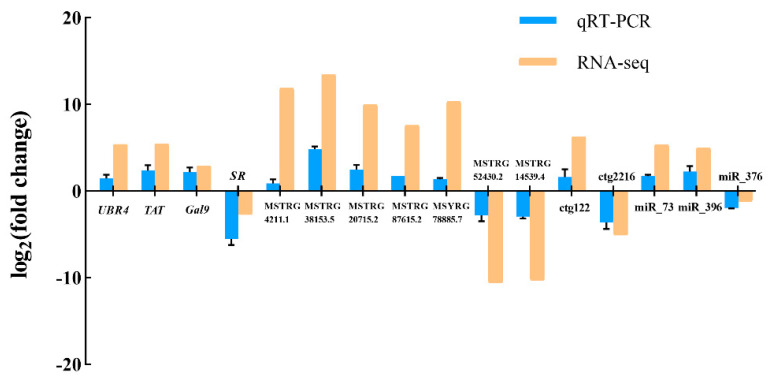
Validation of the RNA-seq data by qRT-PCR. The four on the left are DEGs, the next seven are DELs, the next two are DECs, and the three on the right are DEMis. The qRT-PCR data presented are the means ± SD of three biological replicates, and each measurement was repeated three times.

As shown in Figure 6, qRT-PCR results revealed that the expression of 16 selected RNAs including four mRNAs, seven lncRNAs, two circRNA, and three miRNAs were in good agreement with the transcriptome analysis results, demonstrating good the reliability of whole transcriptome sequencing results.

### 3.8. Effect of Salinity Stress on the Expression of Related Genes

**Figure 7:**
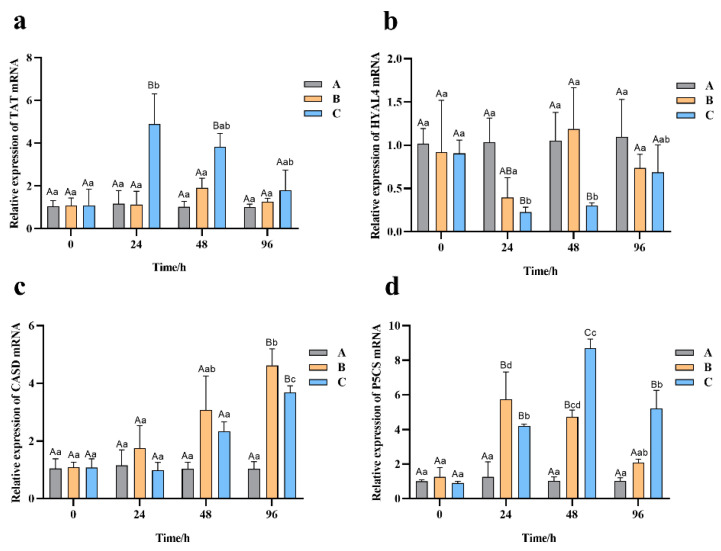
The expression pattern of four candidate genes involved in osmotic pressure regulation under high salinity stress. (**a**) *TAT*, (**b**) *HYAL4*, (**c**) *CSAD*, and (**d**) *P5CS* expression levels. Different capital letters represent significant differences between groups treated with different salinity levels at the same time point (*p* < 0.05), and different small letters represent significant differences between different time points for the same group (*p* < 0.05).

The expression pattern of four candidate genes involved in osmotic pressure regulation under high salinity stress were detected by qRT-PCR. Among them, *TAT*, *CSAD*, and *P5CS* displayed increased and *HYAL4* decreased expression under high salinity in the gills tissue sampled from examined Chinese razor clams (Figure 7).

The *TAT* gene remained stable in group A, fluctuated with no significant difference in group B, and changed significantly in group C with stress time. In group C, the expression of *TAT* increased significantly to the highest level at 24 h, which was about 4 times higher than that of 0 h, and then decreased slowly to the level as in the control group at 96 h (Figure 7a).

In groups A and B, the expression of *HYAL4* was relatively stable with no significant change during treatment time. However, in group C, it decreased sharply from 0 h to 24 h and 48 h (*p* < 0.05) and then returned to the level as in the control group at 96 h (Figure 7b).

In the case of *CSAD* gene, there were no significant difference in its expression among all the groups within 48 h (*p* > 0.05). However, its expression increased significantly in groups B and C at 96 h (*p* < 0.05), with 4.6 and 3.6 times of that in the control group, respectively (Figure 7c).

Figure 7d shows the expression of *P5CS*. In group B, it increased significantly to the highest level at 24 h, which was about 5 times higher than that at 0 h, and returned to the level as in the control group at 96 h. In group C, it increased significantly from 24 h to 48 h and reached the highest level at 48 h, about 8 times higher than that at 0 h, and significantly higher than those of groups A and B at 48 h (*p* < 0.05).

## 4. Discussion

For shellfish living in intertidal areas, estuaries and other environments with varying salinity, rapid physiological adaptation is often necessary. Although the Chinese razor clam is one of the main aquaculture bivalve species in China, it has received little attention in terms of transcriptomic study [40]. In this study, we analyzed the expression profiles of mRNA, lncRNA, miRNA, and circRNA in the gill of Chinese razor clams in response to different salinity levels. Our analysis provided a large number of candidate genes and elements related to osmotic pressure regulation. The results can provide valuable reference data for the study of the regulation mechanism of osmotic pressure in shellfish.

In this study, many GO terms and KEGG pathways enriched by DEGs were involved in response to acute high-salt stress. For example, the taurine and hypotaurine metabolic pathways have been well demonstrated to participate in regulating tissue osmolarity in previous studies [41]. In Chinese razor clams, the content of taurine is reported to increase with salinity [21]. In addition, many other amino acid metabolic pathways including “alanine, aspartate and glutamate metabolism”, “arginine synthesis”, and “tyrosine metabolism” were also significantly enriched. This suggested that free amino acids possibly play important roles in regulating osmotic pressure in Chinese razor clams. Moreover, a variety of immune-related genes, such as galectin, C-type lectin, and C1q domain-containing proteins, in Chinese razor clam gill tissue underwent significant changes upon exposure to high salinity. This is consistent with previous report indicating that exposure to higher salinity can change immune-related gene expression, resulting in immune response [20]. Interestingly, in addition to the previously mentioned *CSAD*, *TAT*, and E3 ubiquitin protein ligases, there was up-regulation in the expression of some genes encoding biological enzymes such as serine hydroxymethyl transferase, glutamate decarboxylase, etc., that play an important role in adaptation to salinity changes [16,42].

LncRNAs are involved in the alteration of the spatial conformation of target gene chromatin, regulation of the target gene expression, and regulation of mRNA post-transcriptional modifications [43,44,45]. The similar GO annotation of DELs and DEGs indicated that lncRNAs and mRNAs might have a potential mutual regulatory relationship. By the KEGG enrichment analysis of DEL target genes, several possible pathways involved in osmotic pressure control were found. For example, “Ubiquitin mediated proteolysis” has been found to play an important role in self-defense, abiotic stress, and other stress resistance in plants [46,47]. In this study, many of the DEL target genes in this pathway were inferred to affect the normal physiological metabolism and increase free amino acid content by decomposing protein under the salinity stress. Interestingly, the KEGG pathway involving the most DEL target genes was “Neuroactive ligand-receptor interaction” which was related to the amino acid metabolism. In a metabolomics study of sea cucumber (*Apostichopus japonicus*), the contents of acetylcholine, glycine, gamma-aminobutyric acid, taurine, L-aspartic acid, L-glutamic acid, adenosine, histamine, and L-glutamate have been found to change significantly in eviscerated and heat-stress individuals, and “Neuroactive ligand-receptor interaction” has been shown to be the enriched pathway [48]. In addition, the ABC transporters was also significantly enriched, and they are involved in regulating the transport of small molecules such as amino acids, ions, and sugars in biological membranes [49]. Therefore, ABC transporters play important roles in maintaining the osmotic balance in cells. These findings contribute to understanding of how lncRNAs are involved in the regulation of hyperosmolarity in Chinese razor clams.

In recent years, a large number of unique circRNAs with important functions have been identified in human and mouse [50]. In contrast, studies on the circRNAs in bivalves are very limited. In the present study, 2890 circRNAs were successfully identified and 182 DECs were screened. These DECs were involved in energy metabolism and signal transduction, which were important physiological processes in response to stress, suggesting the critical role of circRNAs in dealing with high salinity stress in the Chinese razor clam.

Functional studies on miRNAs have revealed that lncRNAs and circRNAs can function as ceRNAs that can derepress genes by adsorption of miRNAs [36]. Although miRNAs have been extensively studied in recent years, they have not been systematically reported in the Chinese razor clams. Importantly, the genes corresponding to DEMis induced by high salinity stress were enriched into the “Hippo signaling” pathway in the current study. This pathway has been shown to play a pivotal role in maintaining tissue homeostasis in multiple organisms ranging from *Drosophila* to mammals and the immune system homeostasis [51]. This suggests that those DEMis may regulate tissue or immune homeostasis by mediating the Hippo signaling pathway during the salinity change period.

The important role of free amino acids in osmoregulation has been widely demonstrated. The most significantly enriched pathway for the differentially expressed RNAs was “amino acid metabolic”. “Glycerophospholipid metabolic” was another significantly enriched pathway, and it has been proved to regulate membranes during osmoregulation in aquatic animals [49]. Therefore, it is possible that under acute high salinity stress, the Chinese razor clam could undergo a series of signal transduction and structural changes of biological membranes through regulating glycerophospholipid and amino acid metabolism. Then, the transport of amino acids, ions, and sugars through the biological membrane is controlled via transporters, finally achieving osmotic pressure equilibrium inside and outside the cell. These results showed that amino acid synthesis and membrane transport were the dominant factors involved in the adaptation of the Chinese razor clam to acute salinity stress, while lipid metabolism and signaling played a supporting role.

The ceRNA networks can show the regulatory relationship among different RNAs. In this study, it revealed that miRNAs could target and regulate genes related to amino acid metabolism, integral component of membrane, etc. For example, seven up-regulated circRNAs and one up-regulated lncRNA were found to target novel_miR_376 and up-regulated *TAT*. In apple (*Malus domestica*), the 5′ end of *TAT* contains cis-acting elements which might be affected by ncRNA [52]. In addition, this study found that 16 down-regulated circRNAs and 13 down-regulated lncRNAs could target novel-miR-396 and novel-miR-73, and down-regulated *GPS2* and *HYAL4*. It has been found that *GPS2* reduces K^+^ channel degradation via lysosomal and proteasomal pathways, thereby regulating biofilm ion channels [53]. Therefore, the downregulation of *GPS2* was supposed to reduce K^+^ transport to the extracellular compartment.

Four critical genes identified from the whole-transcriptome were further verified using samples collected at different salinity challenge time by qRT-PCR. It showed that *TAT* significantly increased after 24 h of treatment at 40 ppt salinity, which was consistent with the trend in transcriptome. Together with studies that the overexpression of *TAT* in apple healing tissues and thale cress (*Arabidopsis thaliana*) can enhance tolerance to drought and osmotic stress [52], it suggested that *TAT* was involved in the Chinese razor clam salt stress regulation. The hyaluronidase (HAYL) can hydrolyze hyaluronan (HA) [54,55] which has water-attracting property and can form gel at higher concentrations. Thus, it can affect water transport and osmotic activity in the extracellular matrix [55]. In this study, down-regulation of *HYAL4* was supposed to reduce HA hydrolysis, which then help maintain water in the cell under external hypertonic environment. The CSAD and P5CS are rate-limiting enzymes for the synthesis of taurine and proline, respectively, and their expression levels affect the contents of the two amino acids. The taurine and proline content can increase when the Chinese razor clam is exposed to high salinity stress [21]. In the present study, the expression of *CSAD* started to increase significantly at 96 h of salinity stress. In Japanese oyster (*Crassostrea gigas*), significant up-regulation of *CSAD* gene at 40 ppt salinity was also recorded, but 7 days from exposure [56]. Similarly, the expression of *P5CS* gene in the Chinese razor clams was also increased with the extension of salinity-stress time, but earlier than that of *CSAD* gene. Nonetheless, the upregulated levels of *CSAD* and *P5CS* probably led to increased taurine and proline levels in the Chinese razor clam’s body under high salinity stress, respectively. The above qRT-PCR results indicate that the expression of these four candidate genes in response to high salinity stress is consistent with the results of transcriptome analysis and previous reports, supporting their important role in the regulation of osmotic pressure in the Chinese razor clam.

## 5. Conclusions

By whole-transcriptome RNA-seq, we identified 83,262 lncRNAs, 52,422 mRNAs, 2890 circRNA, and 498 miRNAs in the Chinese razor clam’s gill tissue. Among them, 2317 lncRNAs, 1531 mRNAs, 182 circRNAs, and 145 miRNAs are differentially expressed among three groups treated with different salinity level. KEGG analysis on the target genes of differentially expressed RNAs revealed a large number of pathways related to the metabolism, signal transduction, and transport, all of which were involved in osmotic pressure regulation, especially the amino acid metabolic pathway. Our results suggest that amino acid synthesis and membrane transport are the main factors involved in the adaptation of Chinese razor clams to increased salinity, while lipid metabolism and signaling plays a secondary role. Constructed ceRNA network consisting of differentially expressed mRNAs, miRNAs, circRNA, and lncRNAs revealed further critical roles of identified by us RNAs in regulation mechanisms responsible for adaptation to high salinity stress of Chinese razor clam. Our study provides new insights into the understanding of the role of ncRNAs in hyperosmotic regulation in the Chinese razor clam.

## Figures and Tables

**Table 1:** Primers used for the qRT-PCR analysis.

Type	Gene Name	Sequences (5′→3′)
mRNA	E3 ubiquitin-protein ligase UBR4 (*UBR4*)	F: TCTGGAGGTACTGGATGGAGCATTC R: GGAGAGGATGGCAGAGTGAGGAG
	tyrosine aminotransferase (*TAT*)	F: TAGCGGAGTCTCTGGGCATACAC R: TGGACGGACTGTTGACAATAATGGC
	galectin-9 (*Gal9*)	F: GGGACTGAGGAGACTGCCATACC R: GTGTTTGTCGTTCACATCCACCTTG
	serine racemase (*SR*)	F: TGTGGAGGTGGAGGACTGGTTG R: GGTTGGTTCCACTGCATACACTCTG
	cysteine sulfinic acid decarboxylase (*CASD*)	F: CCTGACGAGTTGACTGCCATTCTG R: TGACGCTGTACTTGACGATCTTGTG
	∆1-pyrroline-5-carboxylate synthase (*P5CS*)	F: CAAGCAAAAATGAACGG R: CCAAGGGAGAGACCACA
	hyaluronidase 4 (*HYAL4*)	F: ATCAGCAAACAAGCACCGTTCAATC R: CGTATCCAGCAAGGAGAGGTTCAAG
lncRNA	MSTRG.38153.5	F: CAGGGTGGGTGTCCATGATTTAAGG R: CCGTGAAGTGGTTTGCGTGAAATG
	MSTRG.20715.2	F: AAGGGAGGTGCAATGTCGATGTG R: GGACCTCTGCCTTATGTGTTACTGG
	MSTRG.87615.2	F: GCAGAGTCTCTAGCACTGTGTCTTG R: GTCATGGTCAGGCTCGATCACAC
	MSTRG.4211.1	F: GGTGCCGAAGATGCCATCAGTC R: CCTGGTGTGTTAATCGCCTCCTTC
	MSTRG.78885.7	F: AGGACGCTGATTACTCGATTAACGG R: GAGGAGAGTGTGAACTGTGCAAGAC
	MSTRG.52430.2	F: ACCCAGCCCTATGTTGCCATTTG R: TTCTGTCTGTGTGCAGTGATTCTCC
	MSTRG.14539.4	F: AGTCCTACTGGCTGCTGATCCG R: CCTGCTGTGTTTAGACAACCTGGAG
circRNA	ctg122:1490189|1502281	F: TTCAATCCAGCGAACTGCGA R: GTTCGAGAGTTTCGCACGC
	ctg2216:18009|78918	F: GCTGATGTAGTCAGGAAGACGG R: GACGTAGGTCGGGTCATGGA
miRNA	novel_miR_376	F: AATTGTTTGACCGAGGATGGTCA
	novel_miR_73	F: AATCCAGTGACTGGGTGTGGTA
	novel_miR_396	F: AAGCGACCGGTGTCAGGATAA
qRT-PCR of control	Ribosomal protein S9 (*RS9*)	F: TGAAGTCTGGCGTGTCAAGT R: CGTCTCAAAAGGGCATTACC
	U6 snRNA (U6)	F: CTCGCTTCGGCAGCACA R: AACGCTTCACGAATTTGCGT

## Data Availability

All sequencing data was deposited in the NCBI Short Read Archive (SRA) database under the BioProject ID: PRJNA901274. Relevant supporting data can be found within the article and additional files.

## Supplementary Materials

The following supporting information can be downloaded at: https://www.mdpi.com/article/10.3390/biology12010106/s1, Figure S1: Pie chart of sequenced RNAs, Figure S2: Volcano Plot pictures showing log_2_FC and −log_10_Pvalue of mRNA, Figure S3: GO and KEGG pathway analyses of DEGs under salinity stress, Figure S4: Volcano Plot pictures showing log_2_FC and −log_10_Pvalue of lncRNA, Figure S5: GO and KEGG pathway analyses of DELs under salinity stress, Figure S6: Volcano Plot pictures showing log_2_FC and −log_10_Pvalue of circRNA, Figure S7: GO and KEGG pathway analyses of DECs under salinity stress, Figure S8: Volcano Plot pictures showing log_2_FC and −log_10_Pvalue of miRNA, Figure S9: Heat map of all DEMis, Figure S10: GO and KEGG pathway analyses of DEMis under salinity stress; Table S1: KEGG pathway analyses of DEGs under salinity stress, Table S2: Annotation analyses of DEGs under salinity stress, Table S3: KEGG pathway analyses of DELs under salinity stress; Table S4: MiRNA target gene prediction.
