# Supplementary material for: Comprehensive Analysis of Whole-Transcriptome Profiles in Response to Acute Hypersaline Challenge in Chinese Razor Clam Sinonovacula constricta
Source: Biology (Basel). 2023 Jan 10;12(1):106. doi: 10.3390/biology12010106 (PMC9856061; doi:10.3390/biology12010106)
Supplement: Supplementary file 1 [file biology-12-00106-s001.zip › Supplementary information-Figure.pdf]

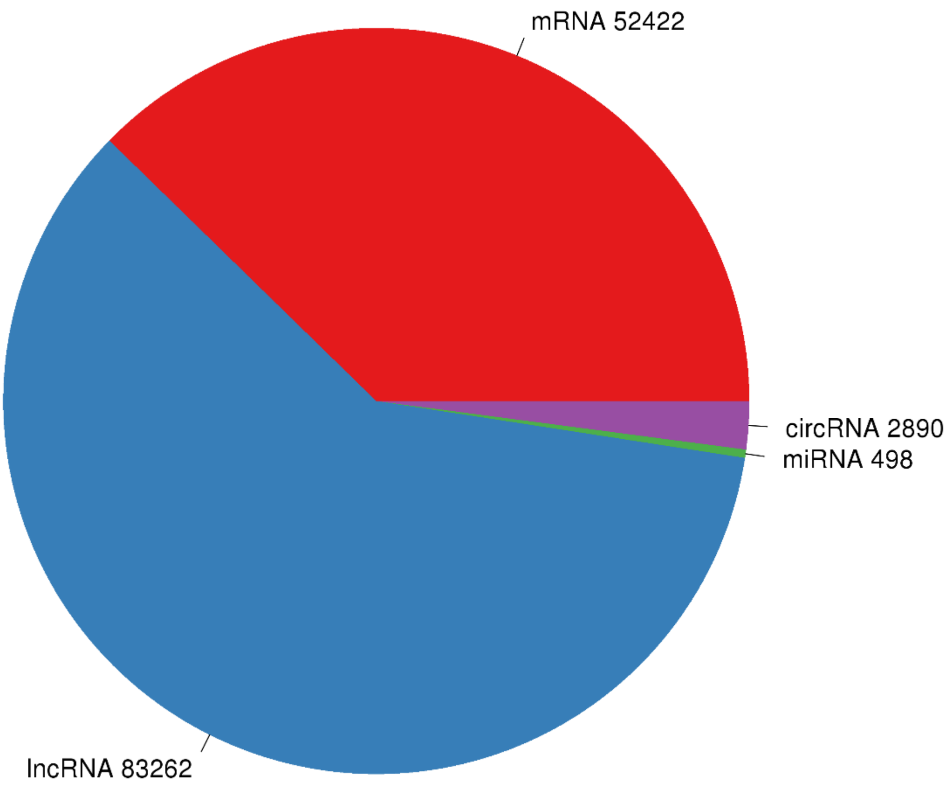

Figure S1. Pie chart of sequenced RNAs.

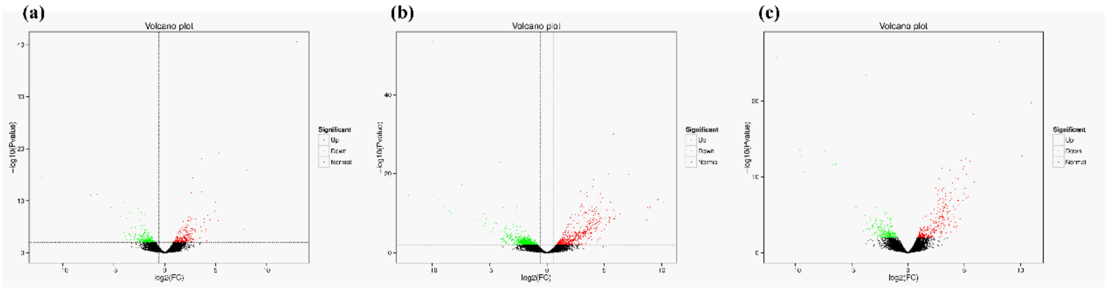

Figure S2. Volcano Plot pictures showing log_2FC and -log_{10}Pvalue of mRNA. (a) Volcano Plot picture in A vs B. (**b**) A vs C and (**c**) B vs C.

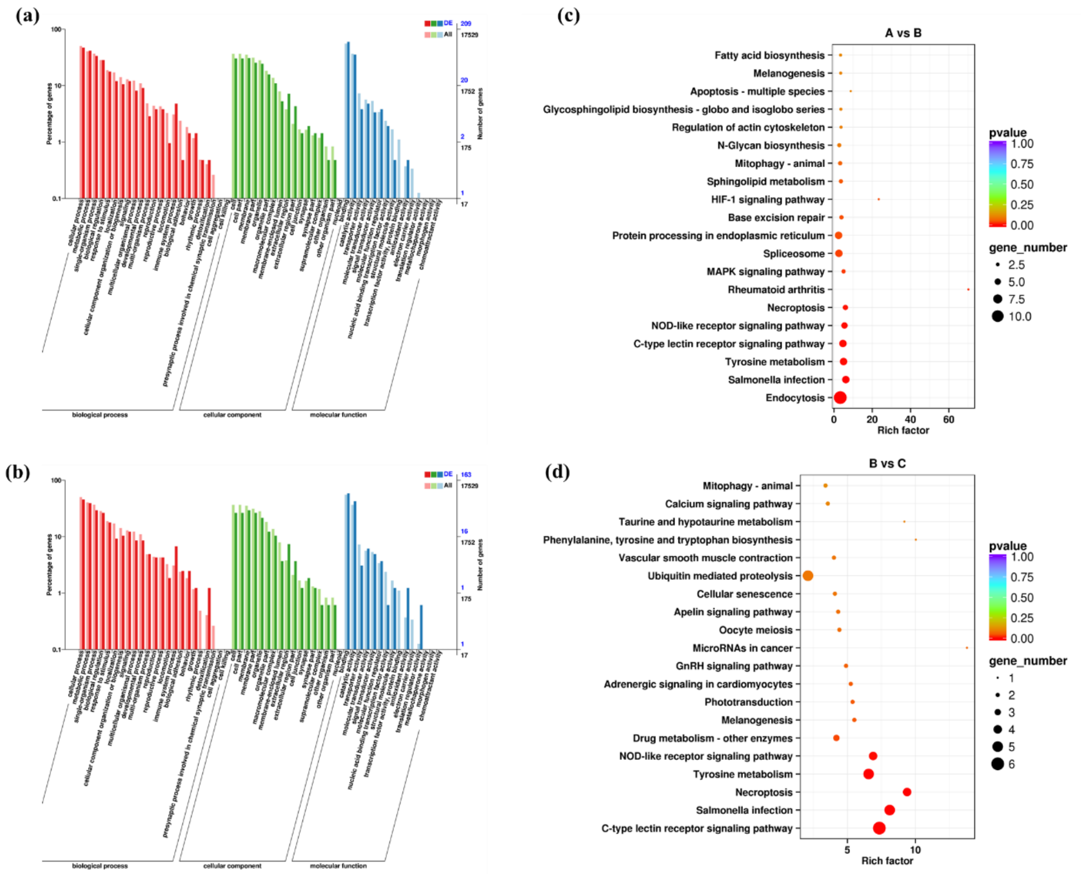

Figure S3. GO and KEGG pathway analyses of DEGs under salinity stress. (**a**, **b**) GO enrichment analysis of DEGs in groups A vs B and B vs C, respectively. (**c**, **d**) KEGG enrichment analysis of DEGs in groups A vs B and B vs C, respectively.

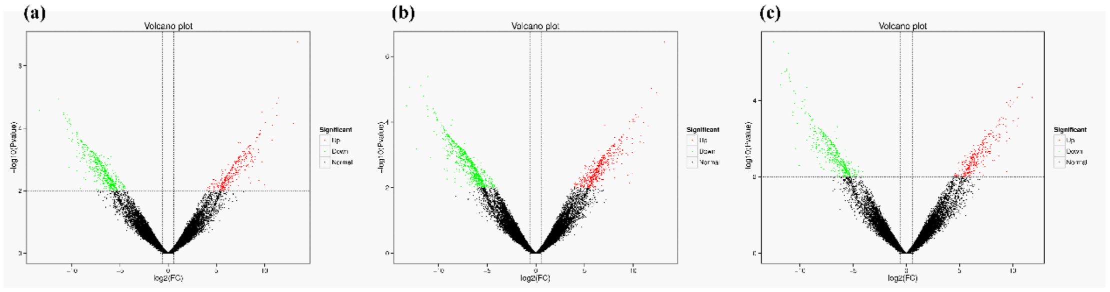

Figure S4. Volcano Plot pictures showing log$_2$FC and -log$_{10}$Pvalue of lncRNA. (**a**) Volcano Plot picture in A vs B. (**b**) A vs C and **c** B vs C.

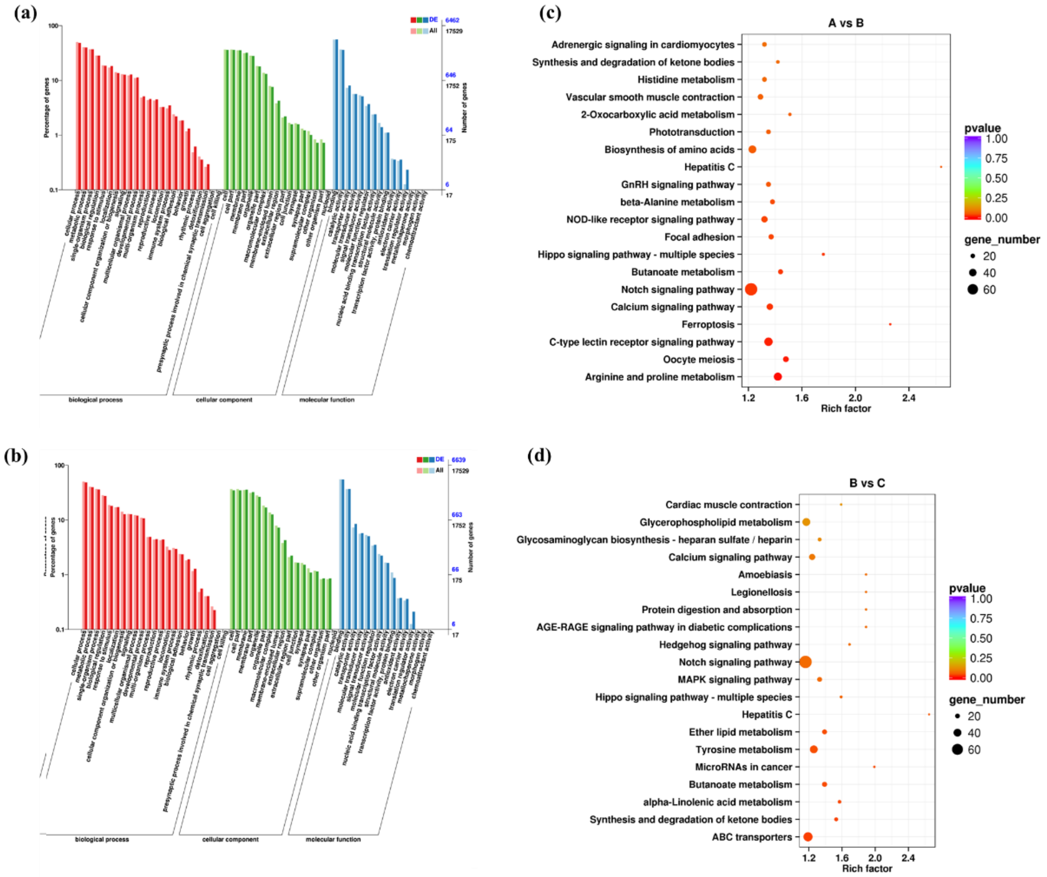

Figure S5. GO and KEGG pathway analyses of DELs under salinity stress. (**a**, **b**) GO enrichment analysis of DELs in groups A vs B and B vs C, respectively. (**c**, **d**) KEGG enrichment analysis of DELs in groups A vs B and B vs C, respectively.

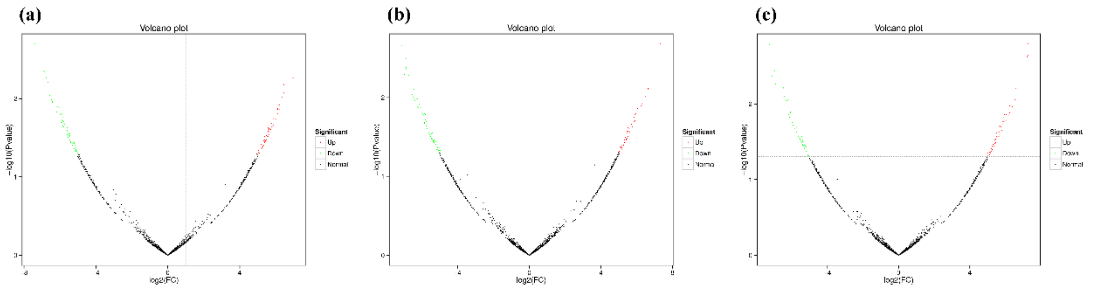

Figure S6. Volcano Plot pictures showing $\log_2$FC and $-\log_{10}$Pvalue of circRNA. (**a**) Volcano Plot picture in A vs B. (**b**) A vs C and **c** B vs C.

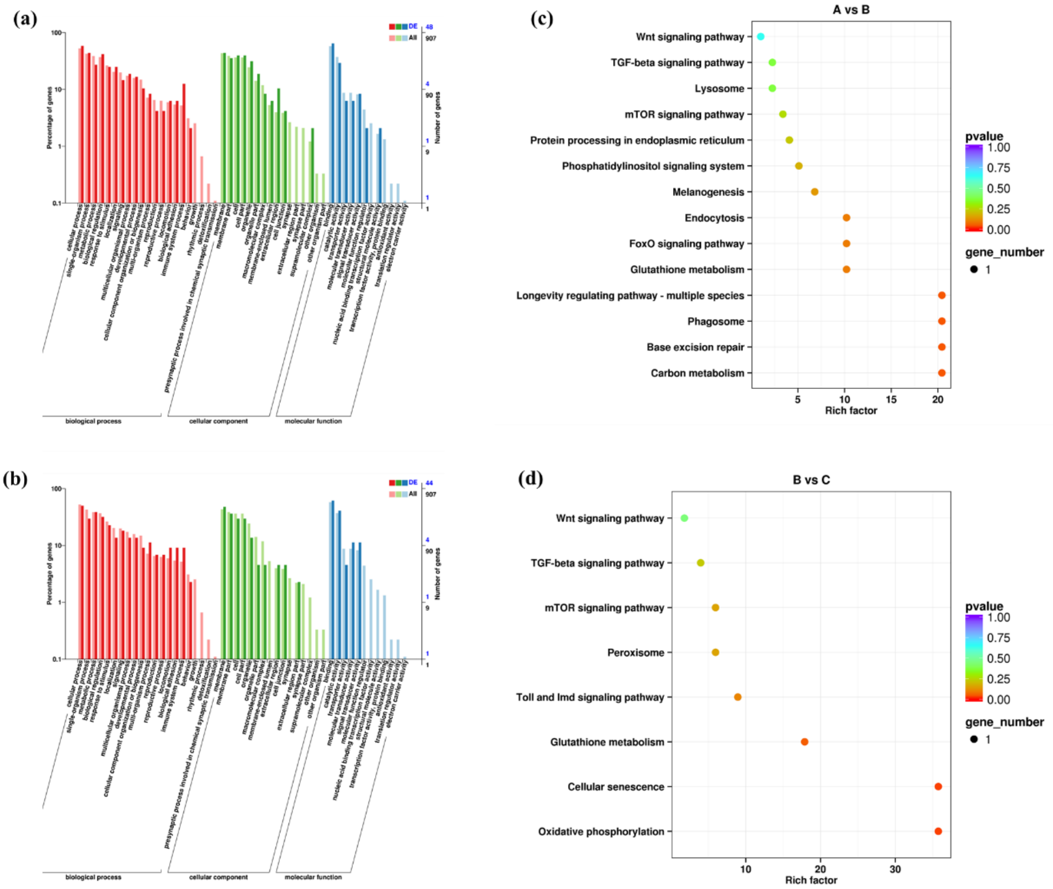

Figure S7. GO and KEGG pathway analyses of DECs under salinity stress. (**a**, **b**) GO enrichment analysis of DECs in groups A vs B and B vs C, respectively. (**c**, **d**) KEGG enrichment analysis of DECs in groups A vs B and B vs C, respectively.

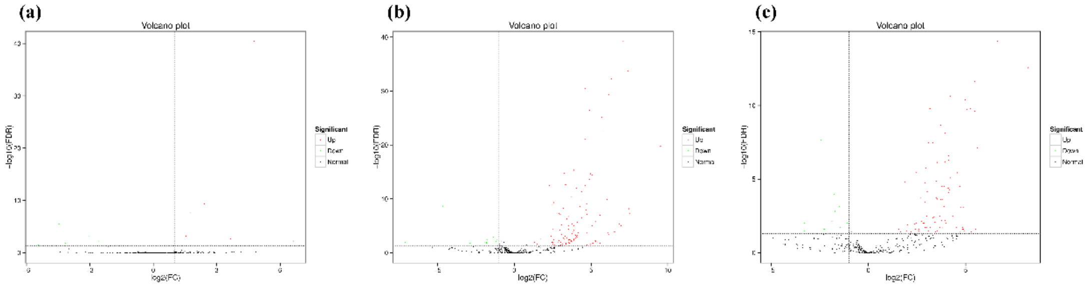

Figure S8. Volcano Plot pictures showing log_2FC and $-log_{10}Pvalue$ of miRNA. (**a**) Volcano Plot picture in A vs B. (**b**) A vs C and **c** B vs C.

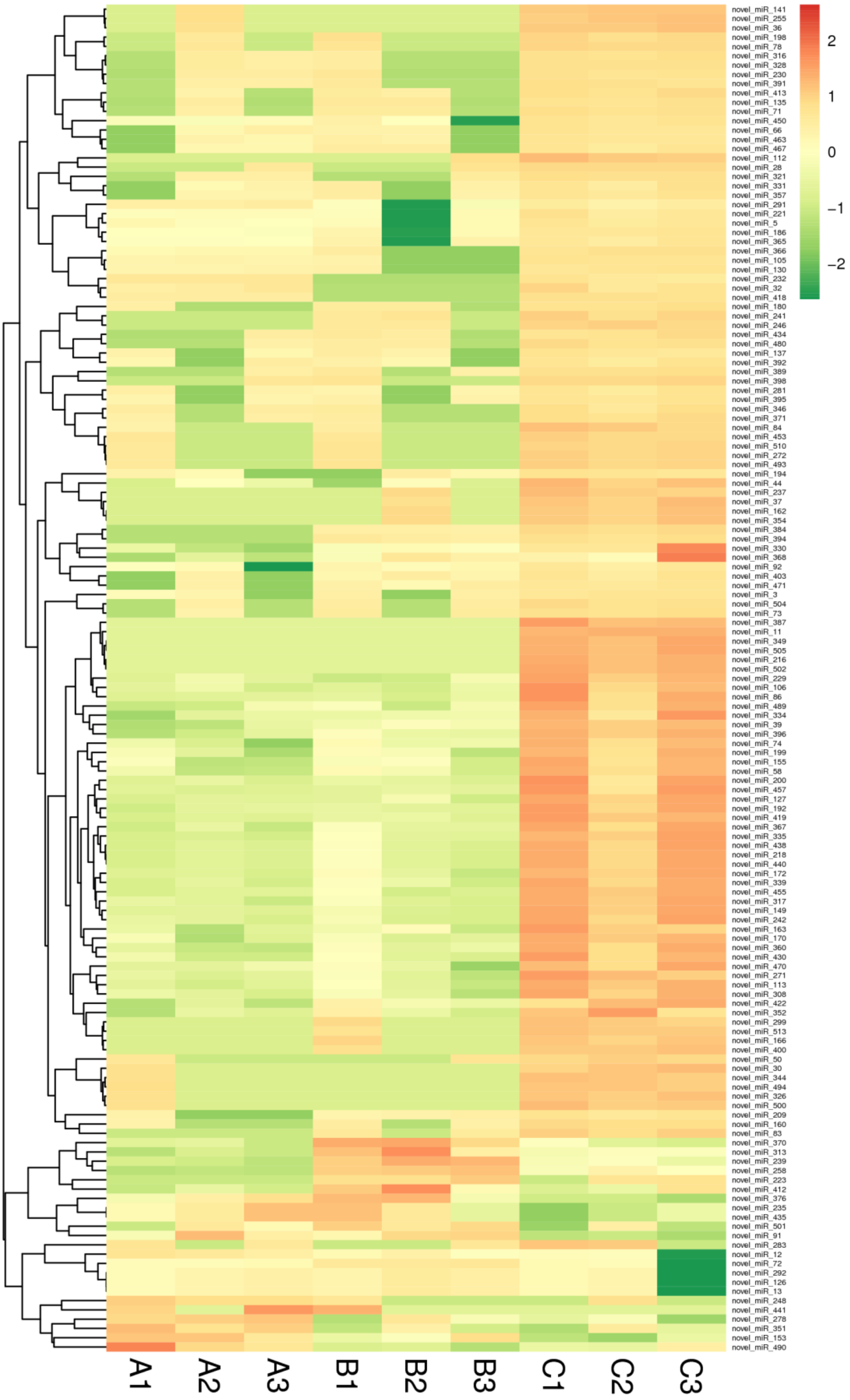

Figure S9. Heat map of all DEMis.

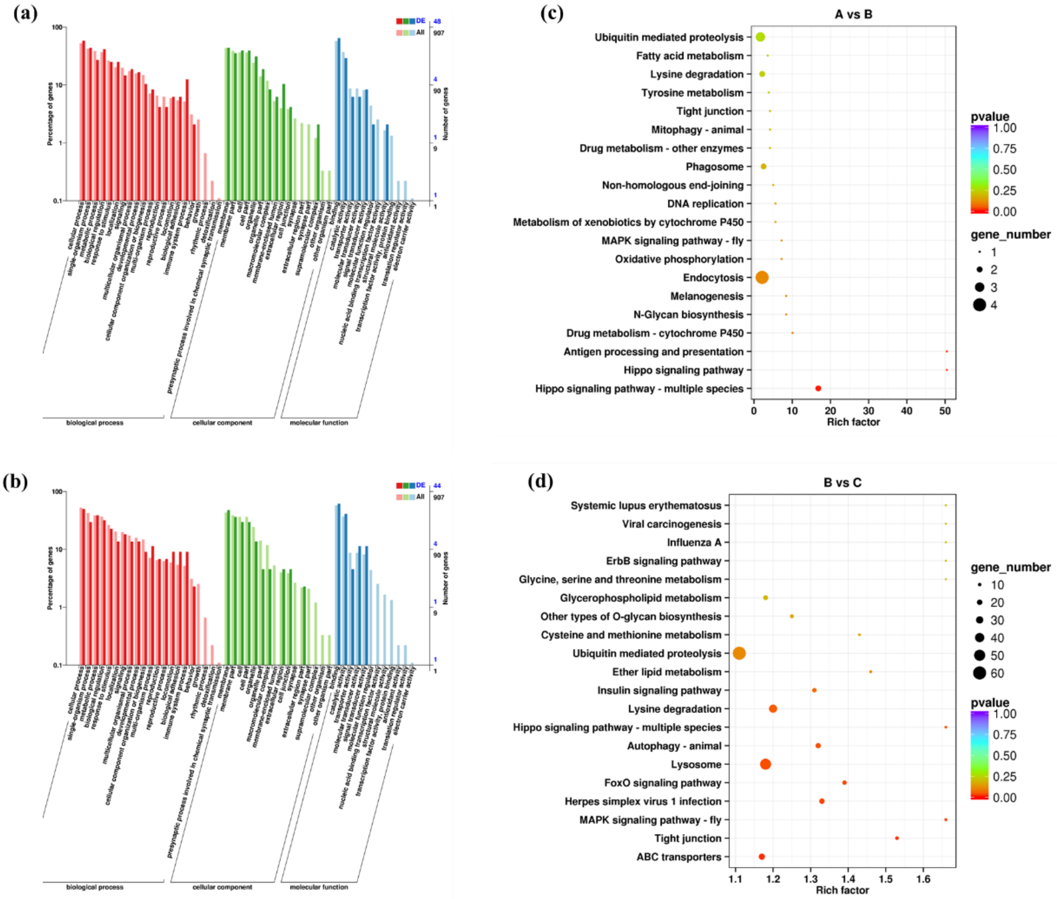

Figure S10. GO and KEGG pathway analyses of DEMis under salinity stress. (**a**, **b**) GO enrichment analysis of DEMis in groups A vs B and B vs C, respectively. (**c**, **d**) KEGG enrichment analysis of DEMis in groups A vs B and B vs C, respectively.
